# Metabolic Syndrome and Its Components in Patients with COVID-19: Severe Acute Respiratory Syndrome (SARS) and Mortality. A Systematic Review and Meta-Analysis

**DOI:** 10.3390/jcdd8120162

**Published:** 2021-11-25

**Authors:** Sergio Rico-Martín, Julián F. Calderón-García, Belinda Basilio-Fernández, María Zoraida Clavijo-Chamorro, Juan F. Sánchez Muñoz-Torrero

**Affiliations:** 1 Department of Nursing, Nursing and Occupational Therapy College, University of Extremadura, 10003 Cáceres, Spain; sergiorico@unex.es (S.R.-M.); zoraidacc@unex.es (M.Z.C.-C.); 2 Nursing Departament, University Center of Plasencia, University of Extremadura, 10600 Plasencia, Spain; bbasfer@unex.es; 3 Department of Internal Medicine, Hospital San Pedro Alcántara, 10001 Cáceres, Spain; juanf.sanchezm@gmail.com

**Keywords:** COVID-19, metabolic syndrome, obesity, diabetes, hypertension, mortality, severe acute respiratory syndrome

## Abstract

Recent meta-analysis studies have reported that metabolic comorbidities such as diabetes, obesity, dyslipidaemia and hypertension are associated with higher risk of severe acute respiratory syndrome (SARS) and mortality in patients with COVID-19. This meta-analysis aims to investigate the relationship between metabolic syndrome (MetS) and its components with SARS and mortality in COVID-19 patients. Methods: A systematic search was conducted in the several databases up until 1 September 2021. Primary observational longitudinal studies published in peer review journals were selected. Two independent reviewers performed title and abstract screening, extracted data and assessed the risk of bias using the Newcastle–Ottawa Scale. Results: The random effects meta-analysis showed that MetS was significantly associated with SARS with a pooled OR (95% CI) of 3.21 (2.88–3.58) and mortality with a pooled OR (95% CI) of 2.32 (1.16–4.63). According to SARS, the pooled OR for MetS was 2.19 (1.71–2.67), *p* < 0.001; significantly higher than the hypertension component. With regard to mortality, although the pooled OR for MetS was greater than for its individual components, no significant differences were observed. Conclusions: this meta-analysis of cohort studies, showed that MetS is better associated to SARS and mortality in COVID-19 patients than its individual components.

## 1. Introduction

COVID-19 is an infectious viral disease that emerged in Wuhan (China) at the end of 2019 and is currently a major cause of mortality across the globe [1]. Approximately 33% of patients hospitalised with COVID-19 develop severe pneumonia requiring admission to intensive care units [2]. Recent meta-analysis studies have reported that metabolic comorbidities such as diabetes [3,4], obesity [5], dyslipidaemia [6] and hypertension [7] are associated with a higher risk of severe acute respiratory syndrome (SARS) and mortality in patients with COVID-19. 

Metabolic syndrome (MetS) is defined by a clustering of five risk factors for cardiovascular morbi-mortality [8], including elevated blood pressure, dyslipidaemia (triglycerides and HDL-cholesterol disorder), glucose metabolism disorders and obesity (at least three factors must be present in order to have MetS). Currently, three cohort studies have reported the association between metabolic syndrome and its components with mortality in patients with COVID-19 [9,10,11], and four cohort studies with SARS [9,10,12,13]. However, no meta-analysis analysing the relationship between MetS and its components with SARS and/or mortality has been published. Therefore, this meta-analysis aims to investigate the relationship between MetS and its components with SARS and mortality in COVID-19 patients.

## 2. Methods

This systematic review and meta-analysis, including data on more than 2400 subjects, was performed according to the Preferred Reporting Items for Systematic Reviews and Meta-Analyses (PRISMA) statement [14].

A systematic search was conducted in the Web of Science (WOS), Scopus and PubMed databases up until 1 September 2021. The following keywords were used: “Metabolic syndrome” and “COVID-19” and/or “coronavirus”. All articles with English or Spanish abstracts and full texts were evaluated. No additional filters were applied. Two independent reviewers (J.F.C-G and J.F.S.M-T) conducted title and abstract screening, extracted data and assessed the risk of bias using the Newcastle–Ottawa Scale [15]. Discrepancies were resolved by agreement with the third reviewer (S.R-M). The inclusion criteria used were longitudinal observational studies published in peer review journals that reported the association between MetS and its components with SARS and/or mortality in COVID-19 patients. 

Articles reporting the odds ratio (OR) and its 95% confidence interval (95% CI) were included in the meta-analysis. Where articles gave values adjusted for covariates, these values were preferred to unadjusted values. The pooled effect size and their 95% CI to estimate the association between MetS and its components with SARS and mortality was calculated using the inverse variance method. OR for each article were pooled using the average value and standard error (SE). SEs were calculated with this measure (SE = upper limit of 95% CI − OR/1.96). Heterogeneity was assessed using the I^2^ statistic, which was interpreted accordingly as follows: modest (0–25%), moderate (25–50%), substantial (50–75%), and considerable (75–100%). We estimated a random-effect model when substantial to considerable heterogeneity was present, and a fixed-effect model was used when there was modest or moderate heterogeneity. Random-effects meta-regression models were not conducted because the OR (95% CI) included was adjusted for covariates. We evaluated the publication bias by Egger’s test. All analyses were conducted using Review Manager software (RevMan V.5.3.5), and we considered *p* < 0.05 statistically significant. 

## 3. Results

### 3.1. Study Selection

**Figure 1:**
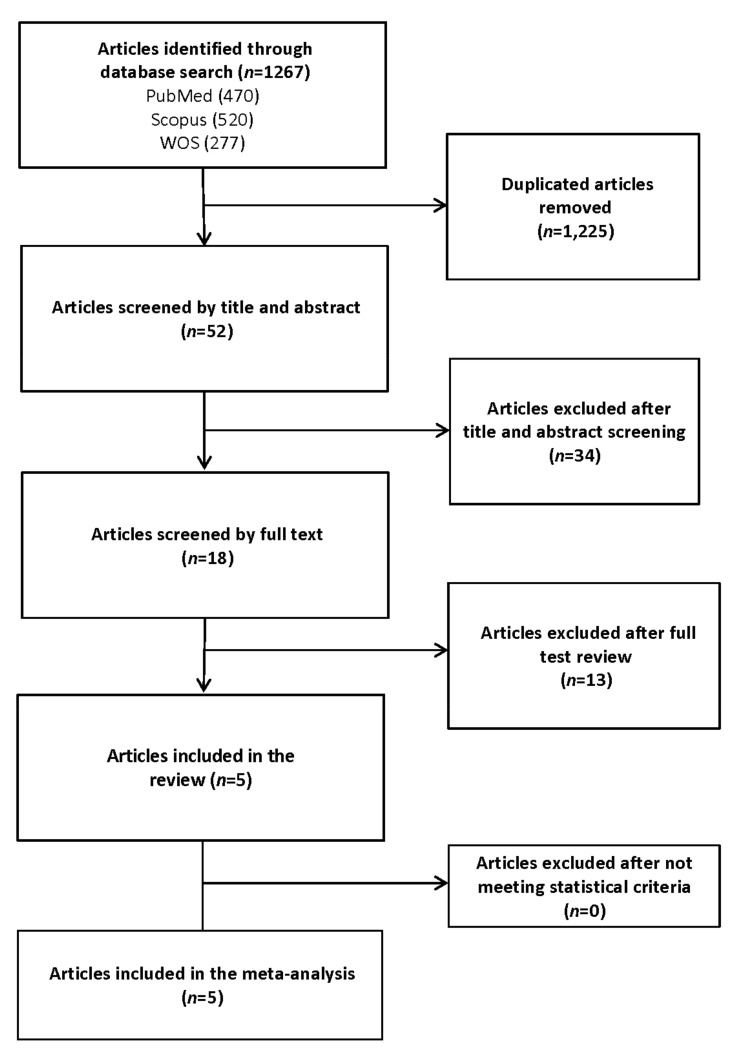
Search strategy to identify articles on the relationship between metabolic syndrome (MetS) and its components with severe acute respiratory syndrome (SARS) and/or mortality in patients with COVID-19.

The systematic search detected 1277 references through keyword search, including 277 papers from WOS, 470 from PubMed, and 520 from Scopus. Of these, 1225 were duplicates, obtaining 52 papers for the title and abstract evaluation. After titles and abstracts revision, 34 articles were excluded. Eighteen studies were selected for review after full-text evaluation. Of these, 13 papers were excluded. Therefore, five articles fulfilled the inclusion criteria and were incorporated in the systematic review and meta-analysis. The study selection process is illustrated in Figure 1. 

### 3.2. Study Characteristics

Five studies were included in this systematic review. Of these, four assessed the association of MetS and its components with SARS, and three with mortality. Four studies were retrospective cohort studies and one was a prospective cohort study. All included articles in this systematic review were published between 2020 and 2021. These studies were performed in four countries including the United States (*n* = 2), Iran (*n* = 1), the Netherlands (*n* = 1) and Israel (*n* = 1). The number of participants ranged considerably (from 71 to 1871) between the articles with a median of 157 and an average of 493. According to population characteristics, one study evaluated patients that were admitted to intensive care units. A total of four studies adjusted their results for health-related characteristics (e.g., age, sex, lung diseases, smoking status, etc.). Basic study characteristics included in the review are shown in Table 1.

### 3.3. Meta-Analysis

We conducted a meta-analysis to examine the association between MetS and its individual components with SARS and mortality in COVID-19 patients. Three studies [9,10,11] were included for mortality outcome, and four studies [9,10,12,13] for SARS outcome (MetS: 4 studies [9,10,12,13]; diabetes and obesity: 3 studies [9,12,13]; hypertension and hyperlipidaemias: 2 studies [9,12]).

**Figure 2:**
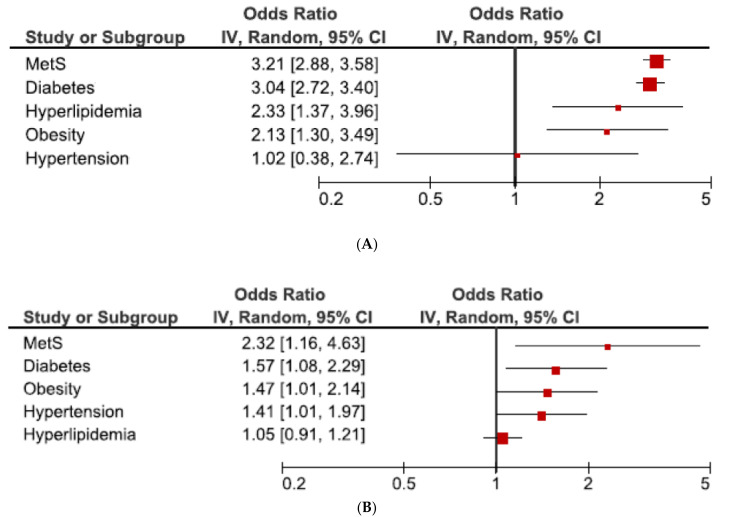
Forest plot of the association between MetS and its components with SARS (**A**) and mortality (**B**) in patients with COVID-19. Abbreviations: CI: confidence interval; IV: inverse variance.

The outcome of the forest plot for the connection between MetS and its component with SARS and mortality of patients with COVID-19 is presented in Figure 2A,B. In a random effects meta-analysis, our results showed a close relationship between MetS and SARS, with a pooled OR (95% CI) of 3.21 (2.88–3.58). Considerable heterogeneity was identified among studies (I^2^ = 83%; *p* < 0.001). In addition, the estimated pooled OR for diabetes, hyperlipidaemia and obesity, but not hypertension, were significantly associated with SARS. The pooled OR (CI% 95) difference between MetS and hypertension was 2.19 (1.71–2.67); *p* < 0.001. The estimated pooled OR for diabetes: 3.04 (2.72–3.40) I^2^ = 65%, hyperlipidaemia: 2.33 (1.37–3.96) I^2^ = 0%, and obesity: 2.13 (1.30–3.49) I^2^ = 99% were lower the pooled OR for MetS; however, no significant differences were found. 

According to mortality (Figure 2B), pooled OR for MetS and its individual components (diabetes, hypertension and obesity, except hyperlipidaemia) were significantly associated with mortality. In this meta-analysis, pooled OR for MetS was greater that pooled OR for individual components (diabetes, obesity, hypertension and hyperlipidaemia); however, no significant differences were observed.

### 3.4. Quality of Studies and Publication Bias

Table 1 shows the assessment results of the quality of the studies included according to NOS. All studies had a score ≥ 7 points. The mean score was 7.8 out of 9 (range from 7 to 8). Egger’s test showed no publication bias (*p* > 0.1).

## 4. Discussion

The main finding of this meta-analysis has shown that COVID-19 patients with MetS presented greater OR for SARS and mortality that COVID-19 patients without MetS but with presence of some of its components (diabetes, hyperlipidaemia, obesity or hypertension).

Several systematic reviews and meta-analysis have concluded that metabolic comorbidities such as diabetes [3,4], obesity [5], dyslipidaemia [6] and hypertension [7] are associated with higher risk of severe acute respiratory syndrome (SARS) and mortality in patients with COVID-19. Obesity had previously been related as a risk factor for viral infections due to its effect on the immune response [16,17]. Adipocytes secrete a multitude of factors and hormones that influence many organ systems, including the lungs. Underlying mechanisms of obesity on the severity of COVID-19 may involve abnormalities in the production of adipokines (e.g., increased leptin and decreased adiponectin) by adipose tissue [18]. A balance between adipokines is necessary for an appropriate immune response; however, the imbalance between leptin (pro-inflammatory functions) and adiponectin (anti-inflammatory function) may result in the development of a dysregulated immune response [19]. On the other hand, SARS-CoV-2 has a high affinity to bind the angiotensin-converting enzyme-2 receptors [20], and the visceral adiposity tissues increase its expression, particularly lung tissue [21], increasing susceptibility to COVID-19 [22]. In recent times, other hypotheses have been developed to explain how obesity affects response to infection [18,23]. 

Previous meta-analysis have assessed the association of diabetes, hypertension and other co-morbidities with severity of disease and mortality [24,25]. The findings showed that comorbid diabetes and hypertension have a negative effect on the health status of COVID-19 patients, where diabetes was a greater risk factor than hypertension for severity and mortality. Similar results were observed in our results. Diabetes is considered as the most important cause for mortality in COVID-19 hospitalized patients [3] and has been identified as the second most common comorbidity among cases of COVID-19 [26]. The mechanism underlying the elevated COVID-19 mortality in patients with diabetes may be explained by the increase in chronic inflammatory markers, such as C reactive protein, interleukin 6, interleukin 10, and tumor necrosis factor-α, which promote susceptibility to COVID-19 [27]. On the other hand, it is hypothesized that the worse outcomes of COVID-19 patients with diabetes mellitus are attributable to the angiotensin-converting enzyme-2 receptor-mediated entry of the virus in the host cell, which damages the insulin-producing pancreatic islet cells [28]. Regarding hypertension, the exact mechanism by which hypertension increases mortality rate remains unclear, though chronic inflammation as with diabetes and obesity may play an active role in increasing the risk of death [29]. In addition, the administration of some antihypertensive drugs such as angiotensin receptor blockers or angiotensin-converting enzyme inhibitors may also be associated with enhanced angiotensin-converting enzyme-2 receptor expression at the cell surface, increasing susceptibility to COVID-19 [7]. Finally, a meta-analysis has reported that the co-occurrence of diabetes and hypertension increased the risk of severe COVID-19 [30].

Lipids might have implications in the pathophysiology of patients with COVID-19 [31]. The cholesterol in the cell membrane plays an important role when a virus enters the host cell and the efficiency of viral infection is significantly reduced when cholesterol deficiency is induced in the cell membrane [32]. Moreover, when SARS-CoV-2 infection has already occurred, raised LDL-cholesterol levels can promote with macrophages in atherosclerotic plaques and increase the secretion of pro-inflammatory cytokines [33].

This systematic review and meta-analysis has several potential limitations. First, few articles have been included, since few studies have reported the relationship between MetS and mortality or SARS in COVID-19 patients. Second, the finding showed a substantial or considerable level of heterogeneity, and thus should be interpreted with caution. Finally, all studies included in our meta-analyses were observational; consequently, a cause–effect association cannot be inferred. On the other hand, the major strength is that this is the first meta-analysis to study whether the presence of MetS in patients with COVID-19 is associated with a higher risk of SARS or mortality than the presence of any of its components. Our results may have potential implications for daily clinical practice, suggesting early identification of these patients to avoid infection or provide early treatment in the case that they are infected.

## 5. Conclusions

This meta-analysis of cohort studies, including data on more than 2400 subjects, showed that MetS is better associated to SARS and mortality in COVID-19 patients than its individual components. Finally, each component of MetS was related to SARS and mortality, with the exception of hypertension for SARS and hyperlipaemia for mortality.

## Figures and Tables

**Table 1:** Basic characteristics of the studies included in the review.

Ref	Author (Year)	Country	Study Design	Sample Size (% Male)	Age Mean ± SD	Characteristics Population	Dependent Variable	Independent Variable	Adjustment	NOS
1	Xie J et al. (2020)	United States	Retrospective cohort	287 (43.2%)	61.50 ± 15.20	-	Mortality and SARS	MetS, obesity, hypertension, diabetes and hyperlipidaemia	Age, sex, race, hospital site, and Charlson comorbidity index	8
2	Alamdari NM et al. (2020)	Iran	Retrospective cohort	157 (87.9%)	67.43 ± 5.57	ICU admitted	Mortality and SARS ^†^	MetS, obesity, increased blood pressure, increased blood glucose and HDL levels	-	7
3	Lothia P et al. (2021)	United States	Retrospective cohort	1871 (51.6%)	65.25 ± 6.66	-	Mortality	MetS, obesity, hypertension, diabetes and hyperlipidaemia	Age, sex, race, smoking, insurance and comorbidities which include coronary artery disease, congestive heart failure, COPD, asthma, chronic kidney disease, ESRD on dialysis, any malignancy, any liver disease, history of previous stroke, hypertension, diabetes, hyperlipidaemia	8
4	Van Zelst CM (2020)	Netherlands	Prospective cohort	79 (46.8%)	59.50 ± 16.44	-	SARS	MetS, obesity, hypertension, diabetes and hyperlipidaemia	Age, sex, MetS, waist–hip ratio and BMI	8
5	Mahamid M (2020)	Israel	Retrsopective cohort	71 (28.2%)	51.00 ± 71.80	-	SARS	MetS, obesity, diabetes	NAFLD, obesity, hypertension, metabolic syndrome, diabetes and smoking	8

Abbreviation: BMI, body mass index; COPD, chronic obstructive pulmonary disease; ESRD, end stage renal disease; HDL, high-density lipoprotein; MetS, metabolic syndrome; NAFLD, nonalcoholic fatty liver disease; NOS, Newcastle–Otawa Scale; ICU, intensive care unit; IMV, invasive mechanical ventilation; SARS, severe acute respiratory syndrome. † Only for MetS.

## Data Availability

Not applicable.
